# “Available upon request”: not good enough for microbiome data!

**DOI:** 10.1186/s40168-017-0394-z

**Published:** 2018-01-10

**Authors:** Morgan G. I. Langille, Jacques Ravel, W. Florian Fricke

**Affiliations:** 1 Department of Pharmacology, Dalhousie University, Halifax, NS Canada; 2 Institute for Genome Sciences, University of Maryland School of Medicine, Baltimore, MD USA; 3 Department of Nutrigenomics, University of Hohenheim, Stuttgart, Germany

Open data that is free and publicly available without restrictions is critical for progress in any scientific discipline and has been the cornerstone of sound and reproducible genomics research. Microbiome research is still a relatively young, thriving, active research field, with great biomedical potential. As a large data-driven research field, microbiome projects can include hundreds or even thousands of participants, samples, and associated background (“metadata”) parameters. Processing this data, identifying meaningful associations, and determining significance depends on complex, often non-standardized bioinformatics and biostatistics protocols. Reproducibility, transparency, and expandability of these protocols to review, evaluate, and build upon this work is crucial to fulfill on the promise of microbiome research and maintain credibility. At the absolute minimum, unrestricted access to the raw sequencing data and associated metadata is needed and has been recognized and implemented by the scientific community, some journals, and funding agencies. In practice, access to open protocols for data processing and analysis is also important to promote reproducibility and advances in the field but rarely provided. Unfortunately, there appears to be an increasing number of studies that are failing to satisfy even basic, community-accepted standards.

Motivated by a number of recent negative experiences in our own research projects, as well as our interaction with authors aiming to publish in *Microbiome*, this editorial aims to shed light on common problems in the field and make recommendations to reinforce a culture of open data and protocols for microbiome research.

- Access to sequence data is required by most peer-reviewed journals. However, when we attempted to access published sequence and metadata from microbiome projects, we have often encountered missing, incomplete, inconsistent and/or incomprehensible sequence and metadata, and reluctance by authors, editors, and publishers to react to our complaints.
- Authors increasingly use new models for data distribution, which restrict or limit data access. Data is only made “available upon request” or access granted based on non-transparent, arbitrary, and costly application procedures.
- Reproducibility is further complicated by the limited availability of bioinformatic and biostatistic protocols, including software versions, program parameters, and code of software scripts.

Although personal instances will vary, examples like the one highlighted in Table 1 are commonplace and largely unreported. We believe that the field would greatly benefit from an improved open data and open protocol culture. In the following, we outline a number of recommendations, which we have begun implementing at *Microbiome*:

- Free unrestricted access to data and metadata, non-commercial bioinformatic software, options and code of published analysis should be given at the time of manuscript peer review and ongoing once published.
- Released data and protocols should encompass all parameters and analyses (including the code and scripts used) that are part of the publications and needed to fully reproduce its results.
- Journal peer review guidelines should be extended to include checking compliance with open data and protocol guidelines.
- Journal responsibilities should be extended and reinforced to control compliance and to react to non-compliance.

**Table 1:** Personal experience

The following example was picked, because it represents a high-profile microbiome project with one of the most extensive collections of microbial sequence and health-related human background data to date [1]. As such, it could be a tremendous resource for extended research by the scientific community and has been of interest to on-going projects by the authors of this editorial.
Instead of simply obtaining the data through direct download from one of the existing publicly funded repositories, we were forced to undertake several time-consuming tasks. Here are the steps we took to obtain a particular dataset before eventually giving up:
1. Sent an email requesting the data and our intended use of the data.
• Wait 1 month for response.
2. Obtained response indicating that we need to first fill out a three-page form including what data we want, the title of our project, a summary of the research proposal, our expertise in analyzing this data, and a recent publication record.
• Wait 2 months for approval.
3. Were then sent a “Data Transfer Agreement” that needs to be signed by our institution.
• Wait 2 weeks for reply from institution.
4. Were asked to provide a copy of ethical approval for our project, which we do not have and would not need if the data were publicly available.
• Instead of waiting yet another month for ethics approval, we decide to abandon this dataset for our scientific plan.

We are concerned that recent trends will continue and that they will set the precedent for data access restriction, greatly limiting scientific progress and reproducibility. We should note that some may try to contest open data access under the veil of privacy, but while data must be handled ethically, the public release of non-identifiable molecular data that has already led to publishable results must be the *minimum* moral/scientific standard to which researchers must be held. Further, funding agencies (public and private) should require their grantees to be fully compliant with open data access policies and endorse open data guidelines developed by the scientific community. We would encourage all microbiome researchers including authors, editors, and peer reviewers to stand up for open data access in order to ensure progress, credibility, and reproducibility in this rapidly developing research field.
